# Relationship between white matter integrity and serum inflammatory cytokine levels in drug-naive patients with major depressive disorder: diffusion tensor imaging study using tract-based spatial statistics

**DOI:** 10.1038/s41398-018-0174-y

**Published:** 2018-08-01

**Authors:** Koichiro Sugimoto, Shingo Kakeda, Keita Watanabe, Asuka Katsuki, Issei Ueda, Natsuki Igata, Ryohei Igata, Osamu Abe, Reiji Yoshimura, Yukunori Korogi

**Affiliations:** 1 Department of Radiology, University of Occupational and Environmental Health, Kitakyushu, Japan; 2 Department of Psychiatry, University of Occupational and Environmental Health, Kitakyushu, Japan; 3 Department of Radiology, Graduate School of Medicine, The University of Tokyo, Tokyo, Japan

## Abstract

Recently, accumulated evidence has indicated a role of inflammation in the pathogenesis of major depressive disorder (MDD). Therefore, we evaluated the relationship between white matter integrity and serum cytokine levels during the first depressive episode in drug-naive MDD patients, using a tract-based spatial statistics (TBSS) method. A total of 35 drug-naive MDD patients with a first depressive episode and 35 healthy subjects (HS) underwent diffusion tensor imaging, and an analysis was conducted using TBSS. We measured serum cytokine levels (interleukin [IL]-1β, IL-6, interferon-γ, and tumor necrosis factor-α). Fractional anisotropy (FA) values of the bilateral inferior fronto-occipital fasciculus (IFOF) and genu of the corpus callosum in MDD patients were decreased significantly to the HS (*p* *<* 0.05 with family-wise error [FWE] correction) and were significantly inversely correlated with the IL-1β levels (*p* *<* 0.05, with FWE correction). No regions showed a correlation between FA values and other serum cytokine levels. Our results suggested that the microstructural changes in IFOF and genu of the corpus callosum are associated with the high IL-1β levels in the early stage of MDD.

## Introduction

Quantitative measures by Diffusion tensor imaging (DTI) such as fractional anisotropy (FA) values are useful markers for microstructural changes in white matter (WM). Reduced FA values are thought to reflect reduced organization of the WM, reduced axonal density, and/or reduced myelination^1,2^. Many previous DTI studies have reported alterations (i.e., reduced FA values) in various WM fiber tracts among major depressive disorder (MDD) patients^3–11^. However, the pathogenesis of WM alteration in MDD is only partly understood.

Recently, accumulated evidence has indicated a role of inflammation in the pathogenesis of MDD. For example, some studies have detected higher levels of inflammation in MDD patients than in healthy subjects (HS), although the strength of the evidence varies according to the type of inflammatory marker (cytokine) that is examined (i.e., interleukin [IL]-1β, IL-6, interferon-γ [IFN-γ], and tumor necrosis factor α [TNF-α])^12–14^. A previous study showed that midlife higher C-reactive protein level, a marker of systemic inflammation, was associated with reduced FA on brain DTI in late life^15^. Another study showed that the increased systemic inflammatory markers (leukocyte early apoptosis) in patients with obstructive sleep apnea correlated with the reduced FA in several brain regions^16^. These evidences suggest that systemic inflammation may affect WM integrity. Therefore, we hypothesized that relatively high levels of systemic inflammation in MDD patients can alter WM integrity, which may play a key role in the progression of the disease to a chronic form^17^. However, to our knowledge, no study has evaluated the relationship between serum cytokine levels and WM integrity in MDD patients, although one magnetic resonance imaging (MRI) study demonstrated a significantly negative correlation between serum proinflammatory cytokine levels and hippocampal volume in MDD patients^18^.

Tract-based spatial statistics (TBSS), a voxel-wise approach, was introduced. TBSS projects subjects’ FA data to a FA tract skeleton and minimizes the effects of misalignment^19,20^. Therefore, we evaluated the relationship between WM alterations and the serum cytokine levels in the first episode, drug-naive MDD patients, using TBSS.

## Materials and methods

### Participants

The protocol of this prospective study using data from a Japanese population was approved by the Institutional Review Board. We obtained written informed consent from all participants after they received a detailed description of the study purpose and design, and possible risks. Most subjects had participated in our previous studies^21,22^, which analyzed the relationship between WM alteration and serum cortisol levels in MDD.

Between March 2009 and January 2014, first-episode and drug-naive patients with MDD were recruited. A psychiatrist (A.K., with 13 years of experience in psychiatry) diagnosed MDD patients using fully Structured Clinical Interview for Diagnostic and Statistical Manual for Mental Disorders, Fourth Edition, Text revision (DSM-IV-TR) Research Version, Non-Patient Edition (SCID-I/NP). To qualify for the study, MDD patients must not have met the criteria for any past DSM-IV-TR Axis I disorder during interviews with the psychiatrist. In short, none of the MDD patients in this study had past episodes of mood disorders.

The severity of depression was evaluated using the 17-item Hamilton Rating Scale for Depression (HAMD-17)^23^. Only those with a total HAMD-17 score of ≥14 (moderate or severe depression) were eligible for the study according to a previous report^24^. The exclusion criteria included any history of neurological diseases or serious physical diseases (hypertension, dyslipidemia, and hyperglycemia) and the presence of other psychiatric disorders (i.e., the subjects exhibited no evidence of schizoaffective disorder, bipolar disorder, Axis II personality disorders, or mental retardation). Therefore, subjects with moderate physical diseases (obesity, hypertension, dyslipidemia, and hyperglycemia) were not excluded. Other exclusion criteria were use of drugs **(**steroid, aspirin, or nonsteroidal anti-inflammatory drugs**)** that affect the immune system. Accordingly, 35 right-handed, first-episode, drug-naive patients with MDD were included (Table 1).

**Table 1:** Demographic characteristics and cytokines levels of participants

	Healthy subjects (*n* = 35)	MDD patients (*n* = 35)	*p*-value	*t*-value	degrees of freedom
Characteristics					
Age, years; mean, (range, SD)	44.0 (20–65, 11.4)	46.3 (20–73, 14.3)	0.463	−7.38	64.9
Female, numbers	13	17	0.469		
Obesity, %	2 (6.1)	2 (6.1)	1.000		
Medical comorbidities					
Hypertension, %	2 (5.7)	8 (22.9)	0.084		
Hyperlipidemia, %	6 (17.1)	12 (34.3)	0.171		
Diabetes mellitus, %	0 (0)	1 (2.9)	1.000		
HAMD-17, mean of total scores (SD)		21.0 (6.0)			
Inflammatory, mean; pg/mL (SD)					
IL-1β	0.058 (0.098)	0.038 (0.044)	0.411	1.094	
IL-6	0.410 (0.364)	0.616 (0.812)	0.152	1.368	
IFN-γ	8.969 (15.225)	10.455 (15.861)	0.111	0.400	
TNF-α	2.336 (6.239)	1.612 (0.598)	0.339	0.684	

Diabetes mellitus defined as a random glucose level of >11.1 mmol/l, fasting blood glucose > 7.0 mmol/l, HbA1c > 6.5%, or the current use of antidiabetic drugs, hypertension (blood pressure > 140/90 mm Hg or current treatment with antihypertensive drugs), dyslipidemia (LDL cholesterol > 3.64 mmol/l, HDL cholesterol 0.91 mmol/l, triglyceride > 1.7 mmol/l, or treatment for dyslipidemia), and obesity (body mass index > 26 kg/m^2^)

*SD* standard deviation, *MDD* major depression disorders, *HAMD-17* 17-item Hamilton Rating Scale for Depression, *IL* interleukin, *IFN* interferon, *TNF* tumor necrosis factor

A total of 35 also were recruited from nearby communities via an interview conducted by the same psychiatrist using SCID-I/NP. The HS consisted of not only staff from our institution but also their relatives (marriage) and close friends. Biologically related relatives were excluded from the HS. No HS had a history of severe medical or neuropsychiatric illness or a family history of major psychiatric or neurological illnesses (Table 1). They also had no history of serious physical diseases and use of drugs **(**steroid, aspirin, or nonsteroidal anti-inflammatory drugs**)**.

A radiologist (S.K., 21 years of experience in neuroradiology) who reviewed the conventional MRI data (including T2-weighted images) reported no gross abnormalities, such as infarcts, hemorrhages, and brain tumors, in any of the study participants.

### Cytokine analysis

Thirty-five human blood samples were assayed in singlicate using the V-PLEX Human Proinflammatory Panel I (4-Plex), a highly sensitive multiplex enzyme-linked immunosorbent assay used to quantitatively measure cytokines, including IL-1β, IL-6, IFN-γ, and TNF-αusing the electrochemiluminescent detection method (MesoScale Discovery, Gaithersburg, MD, USA; Table 1). Mean intra-assay coefficients were <8.5%. Any value that was below the lowest limit of detection (LLOD) for the cytokine assay was replaced with half of the LLOD of the assay. This method is robust and well established^25^.

### MRI acquisition

MRI was performed on the same day as cytokine measurement. Brain imaging examinations were performed with 3 T MRI (Signa EXCITE 3 T; GE Healthcare, Waukesha, WI, USA) with an 8 channel brain coil. Diffusion tensor images were performe with the following parameters: TR/TE = 12000/83.3 ms, slice thickness = 4 mm, no gap, field of view = 26 cm, number of excitations = 1, and spatial resolution = 1 × 1 × 4 mm. Diffusion gradients (b value of 1000 s/mm^2^) were applied simultaneously. The diffusion properties were measured in 25 directions.

### Image processing

The distortion of the diffusion tensor images was corrected by using eddy current correction with the FMRIB Software Library (FSL) v5.0.4. Non-brain tissues were deleted with the brain extraction tool. Voxel-wise statistical analysis was performed using TBSS version 1.1. The FA maps were aligned to a target image as follows: (a) the nonlinear registration of each subject’s FA map was applied into the FMRIB58_FA_1 mm standard-space image as the target image and (b) the target image was affine transformed to the 1 × 1 × 1 mm MNI space (Montreal Neurologic Institute, Montreal, Canada). A mean FA image was created from averaging aligned individual FA images. The FA skeleton representing WM tracts and was created with the threshold of 0.2^19^. Individual FA data were projected to the FA skeleton. The FA, mean diffusivity (MD), radial diffusivity (RD), and axial diffusivity (AD) were created by fitting a tensor model to the raw diffusion data. Subsequently, other relevant DTI output images (MD, AD, and RD) were projected onto the mean FA skeleton so that other diffusivity values could be compared between groups in the same spatial location.

### Statistical analysis

We applied independent samples *t*-tests to assess differences between HS and MDD patients. The chi-squared test was used for gender comparisons.

Voxel-wise statistical analysis was performed using a permutation-based inference tool. FA, MD, AD, and RD values were compared between HS and MDD patients using a two-sample *t*-test. Correlations between the serum cytokine levels and FA values were analyzed using a single-group average with additional covariates. Age and sex were entered as covariates in all voxel-wise statistical analyses using the TBSS. The number of permutations in all voxel-wise analyses was set at 5000. According to previous studies^26,27^, values of *p* *<* 0.05 and >50 voxels were considered to indicate a statistically significant difference and a correlation, after family-wise error (FWE) correction for multiple comparisons at the cluster level, using the threshold-free cluster enhancement option. The anatomical location was detected using the Johns Hopkins University WM tractography atlas and the International Consortium of Brain Mapping DTI-81 WM labels atlas.

Statistical analyses were considered significant if *p* *<* 0.05. Statistical analysis was performed using the free software R, version 3.4.0 (R Statistical and Computing Software; http://www.r-project. org/).

## Results

### Participants

**Fig. 1:**
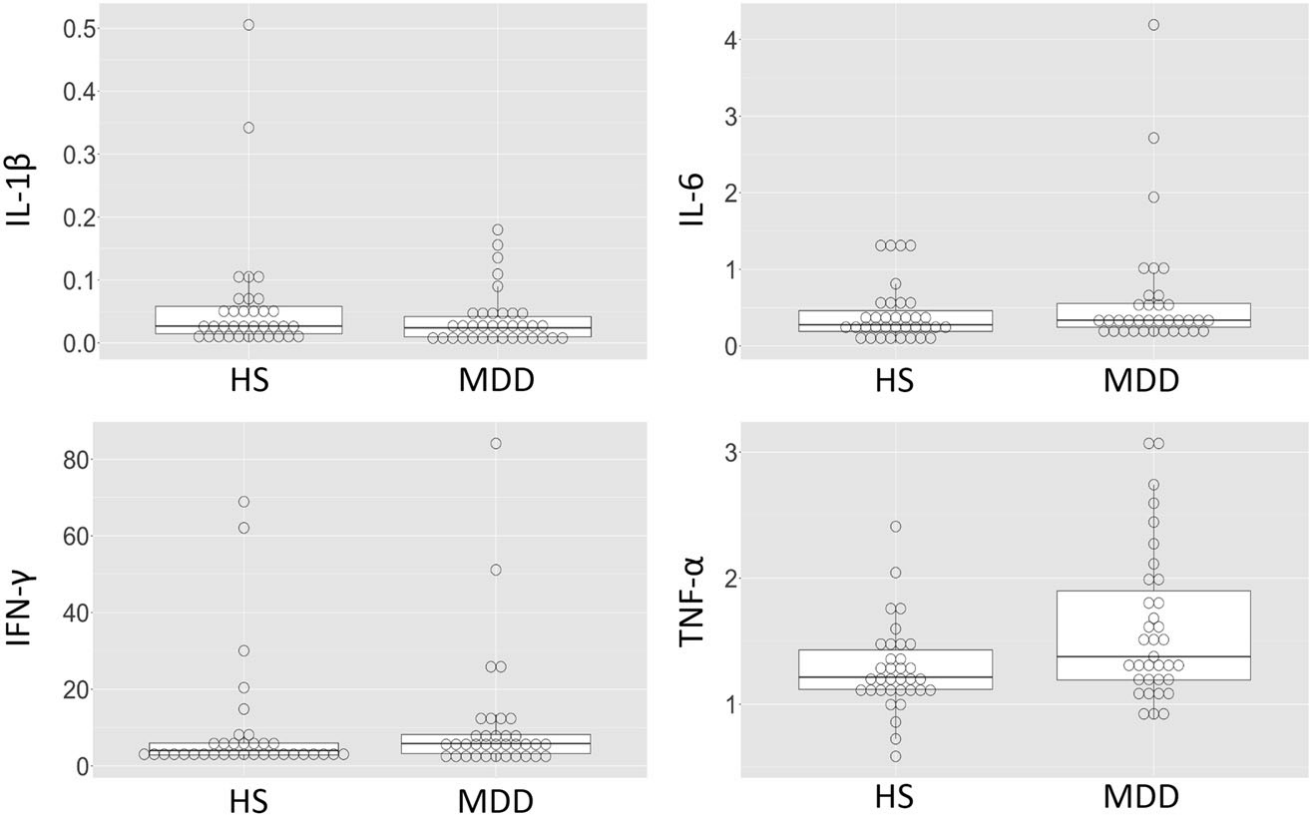
Distribution of cytokine levels Box plots for interleukin IL-1β, IL-6, IFN-γ, and TNF-α levels (HS and MDD). Horizontal lines indicate median values (black lines). The bottom and the top of the box are the first and third quartiles whereas whiskers indicate maximum and minimum values. Outliers are indicated by circles

Table 1 shows the participants’ demographic data. There were no significant differences in age or sex between the HS and MDD patients. For all cytokines, there were no significant differences between the groups (Fig. 1; Table 1).

### Comparison between MDD patients and HS

**Fig. 2:**
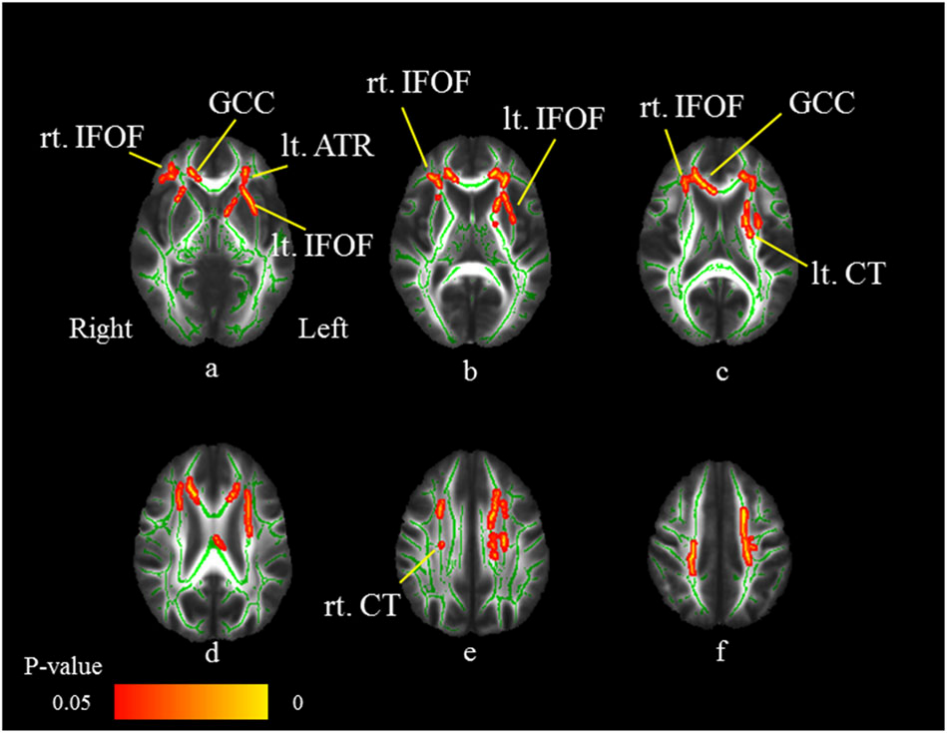
Comparison of FA findings between MDD patients and HS. Axial slices (**a**–**f**) of the cohort’s mean FA skeleton (green) overlaid with red clusters depicting significantly lower FA values in the MDD patients compared to HS (FWE-corrected *p* *<* 0.05). The MDD patients have significantly reduced FA values (FWE-corrected *p* *<* 0.05) in the right corticospinal tract (CT: **e**), right inferior fronto-occipital fasciculus (IFOF: **a**–**c**), left anterior thalamic radiation (ATR: **a**), left CT (**c**), left IFOF, and genu of the corpus callosum (GCC: **a**, **c**)

The cluster voxel size and significant Montreal Neurologic Institute (MNI) coordinates, which are used most widely as a coordinate system, are presented in Table 2. Significant FA differences were observed between the MDD patients and HS. Figure 2 shows the spatial distribution of the brain regions, indicating a reduction of FA values in the MDD patients compared to HS. The MDD patients had significantly reduced FA values (FWE-corrected *p* *<* 0.05) in the right corticospinal tract, right inferior fronto-occipital fasciculus (IFOF), left anterior thalamic radiation, left corticospinal tract, left IFOF, and genu of the corpus callosum (Fig. 2; Table 2). No significant differences were found for the MD, AD, and RD.

**Table 2:** Results of the image analysis

				MNI coordinates, mm		
Anatomical regions	Cluster size	*p*-value^a^	*t*-value	*x*	*y*	*z*
FA values, between-group comparison (control group > MDD group)						
lt. ATR (11.139), lt. IFOF(9.479)	1951	0.033	3.34	114	156	79
lt. CT (7.286)	1427	0.032	3.80	109	108	111
FM (18.098), rt. IFOF (7.698)	1272	0.033	5.44	63	158	90
rt. CT (22.695)	328	0.039	3.92	67	94	114
Negative correlation between FA values and IL-1β levels in the MDD group						
FM (2.741), rt. IFOF (2.567)	27510	0.993	3.43	113	50	92
lt. IFOF (1.127), lt. UF (4.403)	362	0.955	3.91	96	142	61
Interaction of IL-1β level in the control group and MDD group						
lt. ATR (0.591), lt. IFOF(2.588), lt. CT (0.638), rt. CT (1.265), FM (3.624), lt. UF (0.838), lt. ILF (2.588), rt. ILF (1.194)	26781	0.009	2.37	−26	−77	1
lt. ATR (16.101)	574	0.039	3.49	−19	−13	−3

Numbers in parentheses are the probability of fiber tracts

*MNI* Montreal Neuroglogic Institute, *MDD* major depressive disorder, *rt*. right, *lt*. left, *FA* fractional anisotropy, *ATR* anterior thalamic radiation, *CT*C corticospinal tract, *IFOF* inferior fronto-occipital fasciculus, *ILF* inferior longitudinal fasciculus, *FM* forceps minor, including genu of corpus callosum, *UF* unicinate fasciculus

^a^with family-wise error correction

We identified the WM fiber tracts by using the JHU White-Matter Tractography Atlas, except for the genu of the corpus callosum. For identification of the genu of the corpus callosum, we used the International Consortium of Brain Mapping DTI-81.

### Correlation between the cytokine levels and WM integrity

**Fig. 3:**
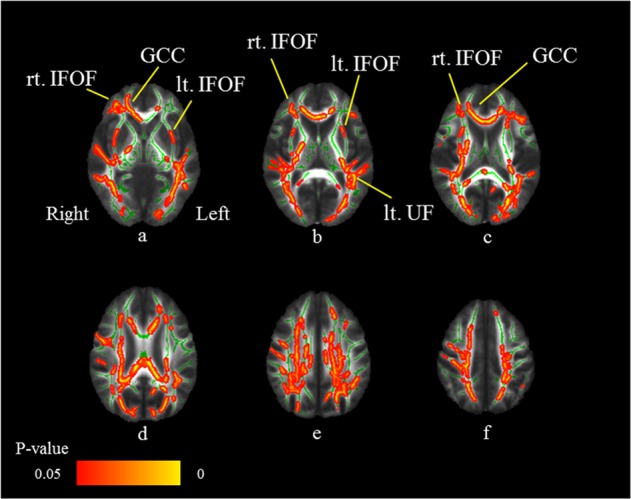
Negative correlations between FA values and IL-1β levels in MDD patients. Axial slices (**a**–**f**) of the cohort’s mean FA skeleton (green) overlaid with red clusters depicting significantly negative correlations between FA values and serum IL-1β levels in the MDD patients (FWE-corrected *p* *<* 0.05). The FA values of the right inferior fronto-occipital fasciculus (IFOF: **a**–**c**), left IFOF (**a**, **b**), left uncinate fasciculus (UF: **b**), and genu of the corpus callosum (GCC: **a**, **c**) reveal significant negative correlations with the IL-1β levels (FWE-corrected *p* *<* 0.05)

**Fig. 4:**
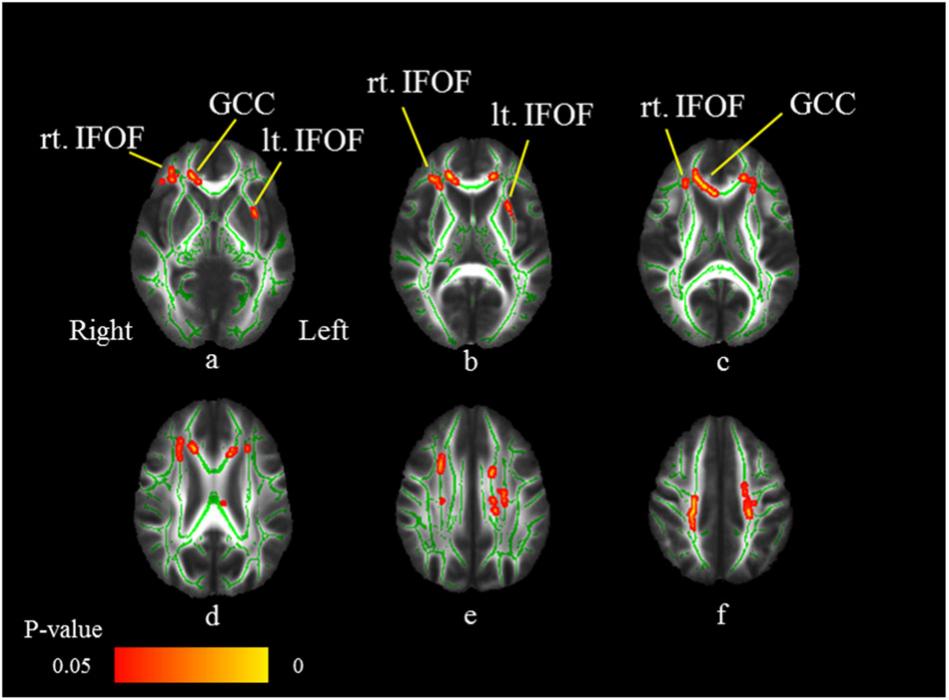
Overlapping voxels of reduced FA values and negative correlations between FA values and IL-1β levels in MDD patients. Axial slices (**a**–**f**) of the cohort’s mean FA skeleton (green) overlaid with red clusters depicting overlapping voxels (bilateral inferior fronto-occipital fasciculus [IFOF]: **a**–**c** and genu of corpus callosum [GCC]: **a**–**c** of the reduced FA values and the negative correlations between FA values and IL-1β levels in MDD patients

Figure 3 shows the areas in which the FA values were associated with the IL-1β levels in MDD patients. The FA values of the right IFOF, left IFOF, left uncinate fasciculus, and genu of the corpus callosum revealed significant negative correlations with IL-1β levels (FWE-corrected *p* *<* 0.05; Table 2). Thus, the FA values of the bilateral IFOF and genu of the corpus callosum in the MDD patients were significantly decreased compared to those in HS and were significantly inversely correlated with IL-1β levels (Fig. 4).

No regions showed a correlation between FA values and serum cytokine levels in the HS and combined subject group (MDD patients and HS).

No regions showed significant correlation between the FA values and other serum cytokine levels. Moreover, no regions showed a positive correlation between FA values and serum cytokine levels. Among the HS, no regions exhibited a significant correlation between FA values and serum cytokine levels.

### Interaction of IL-1β level in MDD patients and HS

**Fig. 5:**
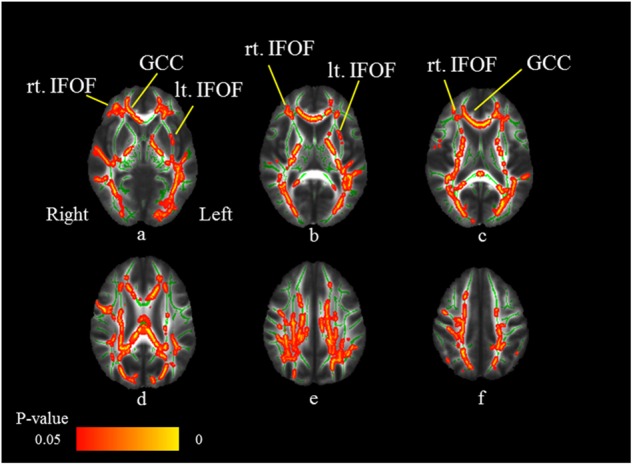
Interaction of IL-1β level in MDD patients and HS. Axial slices (**a**–**f**) of the cohort’s mean FA skeleton (green) overlaid with red clusters depicting interaction of IL-1β level in the HS and MDD patients (FWE-corrected *p* *<* 0.05). Expanded area of white matter fiber tracts, including the bilateral inferior fronto-occipital fasciculus (IFOF: **a**–**c**) and genu of the corpus callosum (GCC: **a**, **c**), show the interaction of IL-1β level in MDD patients and HS (FWE-corrected *p* *<* 0.05)

Expanded areas of WM fiber tracts, including the bilateral IFOF and genu of the corpus callosum, showed the interaction of IL-1β levels in MDD patients and HS (Fig. 5) (FWE-corrected *p* *<* 0.05; Table 2).

## Discussion

Our study provides the first evidence, to our knowledge, of a relationship between microstructural WM alterations and serum cytokine levels in MDD patients. A strength of this study lies in recruitment of first–depressive episode and drug-naive patients with MDD, because according to a meta-analysis, treatment with antidepressants reduces serum cytokine levels in MDD patients^28^. In our study, the FA values of the bilateral IFOF and corpus callosum in the MDD patients were significantly decreased compared to those in HS. In addition, reduced FA values in these WM tracts were correlated with high serum IL-1β levels. These also were supported by results of the interaction analysis. Thus, our results suggested that the neuroinflammatory status in MDD patients is associated with microstructural changes in WM tracts.

In our study, there were no significant differences between the MDD patients and HS for all cytokines, although many studies showed higher serum IL-1β levels in MDD patients than in HS^29,30^. On the other hand, our negative results for serum IL-1β levels may be supported by some previous meta-analyses. Dowlati et al^12^. demonstrated that concentrations of serum IL-1β levels did not differ between 267 depressed and 246 nondepressed subjects. Another meta-analysis also mentioned that patients with MDD compared to controls showed higher levels of IL-6 and TNF-α, but no significant difference in IL-1β levels^17^. Although why serum IL-1β levels were correlated with WM integrity in patients with MDD but not in HS remains unclear, it is important to discuss the possible mechanism underlying these results. We hypothesize that the WM integrity might be more sensitive to IL-1β in MDD patients than in HS, and the microstructural changes in the WM tracts might occur even in the early stage of MDD patients with normal IL-1β level. A recent study by Menard et al.^31^ may support our hypothesis. They investigated effects of chronic social defeat stress, a mouse model of depression, on blood–brain barrier (BBB) permeability and infiltration of serum immune signals. Their results demonstrated that chronic social stress alters BBB integrity through loss of endothelial cell tight junction protein claudin-5, promoting passage of serum cytokines across the BBB. These findings suggest that serum cytokines may easily penetrate the brain parenchyma in MDD patients compared to HS.

Reduced fractional anisotropy values are thought to reflect reduced organization of WM, reduced axonal density and/or reduced myelination^2,32^. Although the exact cause of the reduced FA values in the WM tracts remains unclear in MDD patients, it is important to note that we found that high serum IL-1β levels were correlated with reduced FA values of the IFOF and corpus callosum. The main possible explanation for our result may be due to direct damage to the WM caused by high levels of IL-1β in the MDD brain. IL-1β was an important feature of WM pathology^33^, while another study using ultrasound assessment of WM concluded that cytokine-receptor interactions were more important^34^. IL-1β was localized with matrix metalloproteinase 9 in microglia/macrophages in cerebral areas^33^, indicating that the IL-1 system may be involved in WM damage. Indeed, mice receiving injections of IL-1β showed hypomyelination in the brain, which was characterized by an increased number of nonmyelinated axons and reduced diameter of the myelinated axons^35^. Moreover, these cellular and molecular alterations in WM were correlated with reduced FA values on DTI. Another study demonstrated that intracerebral injections of IL-1β in neonatal rats produced a reduction in the number of developing oligodendrocytes, indicating that direct action of IL-1β impairs myelination^36^. More recently, a rat study by Xie et al^37^. reported that IL-1β also induced hypomyelination in the periventricular WM through inhibition of oligodendrocyte progenitor cell maturation. These evidences support our speculation that WM injury may occur under high serum IL-1β levels in the MDD brain.

Based on the region specificity, our findings may be supported by the results of several DTI studies demonstrating the presence of WM alterations (reduced FA values) in the IFOF^3,4,38^ and corpus callosum^3,7,9,11^ of MDD patients. The IFOF has connections to the inferior and lateral margins of the occipital lobe and to the infero- and dorso-lateral regions of the frontal lobe and is involved in emotional visual function. A previous study has shown that MDD patients have alterations in emotional visual perception, which might be partly attributable to the observed reduced FA values^39^. Moreover, the IFOF is considered to play key roles in the frontal-subcortical circuits. The frontal-subcortical circuits contains five discrete parallel loops that connect specific areas of the frontal cortex through the striatum, globus pallidus, substantia nigra, and thalamus, back to the frontal cortex^40^. Anatomically, the IFOF projects into the frontal cortex from the thalamus. A “disconnection” of the frontal-subcortical circuit was proposed as a pathogenic element associated with MDD^41^. Regarding the WM alterations in the corpus callosum, a recent meta-analysis of DTI studies in MDD patients emphasized the consistent finding of reduced FA values, particularly in the interhemispheric fibers running through the genu and body of the corpus callosum^38^. In addition, a previous study suggests that a history of suicide attempts in mood disorders exerts a specific effect on corpus callosum integrity^42^.

We identified the region (left uncinate fasciculus), wherein the FA values showed significantly negative correlations with IL-1β levels, but no significant differences were noted between the MDD patients and HS. We assumed that in this region, WM may be normal in the early stage of MDD or the WM alterations may lag behind those in the IFOF and genu of the corpus callosum.

Our study has some limitations that should be acknowledged when interpreting the results. First, a small sample of patients was recruited from only one institution, which may represent sampling bias. However, it was difficult to recruit and retain first-episode and drug-naive patients with MDD because many were administered antidepressants before they underwent MRI. Second, the age range was large, which might be associated with heterogeneity of etiology of the sample. Third, in the MDD patients, moderate hypertension, hyperlipidemia, and hypocholesteremia, which might influence the results were not strictly ruled out. Moreover, only patients with a score of ≥14 (moderate or severe depression) on the HAMD-17 were included according to the previous study^24^. Our criteria (excluding 14 or fewer patients) might influence the results. Third, an evaluation of terms of GM damage due to high levels of IL-1β was not included in our study. Using relatively large sample of patients, investigations of the relationship between cortical volume and serum cytokine levels are currently underway in our laboratory. Fourth, we focused on a cross-sectional association based on the one-time assessments of inflammatory markers, although these measurements cannot reliably distinguish between chronic and acute inflammation. The few previous studies that have assessed chronic inflammation have revealed stronger associations with mental health when inflammation is determined using repeated measurements rather than only one measurement^43^. Fifth, we evaluated only the first depressive episode in drug-naive patients with MDD. Therefore, a longitudinal study comprising a larger sample of MDD patients with various conditions is required to determine the causal links between serum IL-1β levels and WM alterations. Finally, we found the nearly significant difference (*p* = 0.05) of the sex ratio between patients and controls, which may affect the results. However, the effect of this bias might be small because age and sex were entered as covariates for all voxel-wise statistical analyses using the TBSS.

In conclusion, in early-stage MDD patients, the FA values of the IFOF and genu of the corpus callosum were significantly decreased compared with those in HS and were significantly inversely correlated with the IL-1β levels. Our results suggested that the microstructural changes in these WM tracts are associated with high IL-1β levels. Given that IL-1β induces hypomyelination in the brain, WM injury might occur under high serum IL-1β levels even in the early stage of MDD.
